# Metabolic profiling shows pre‐existing mitochondrial dysfunction contributes to muscle loss in a model of ICU‐acquired weakness

**DOI:** 10.1002/jcsm.12597

**Published:** 2020-07-16

**Authors:** Paul R. Kemp, Richard Paul, Aaron C. Hinken, David Neil, Alan Russell, Mark J. Griffiths

**Affiliations:** ^1^ Cardiovascular and Respiratory Interface Section, National Heart and Lung Institute Imperial College London, South Kensington Campus London UK; ^2^ Department of Intensive Care Guy's and St. Thomas' NHS Foundation Trust London UK; ^3^ Muscle Metabolism Discovery Performance Unit GlaxoSmithKline, Inc Collegeville PA USA; ^4^ Edgewise Therapeutics Boulder CO USA

**Keywords:** Metabolomics, Muscle wasting, Aortic surgery, Mitochondrial dysfunction, Cortisol

## Abstract

**Background:**

Surgery can lead to significant muscle loss, which increases recovery time and associates with increased mortality. Muscle loss is not uniform, with some patients losing significant muscle mass and others losing relatively little, and is likely to be accompanied by marked changes in circulating metabolites and proteins. Determining these changes may help understand the variability and identify novel therapeutic approaches or markers of muscle wasting.

**Methods:**

To determine the association between muscle loss and circulating metabolites, we studied 20 male patients (median age, 70.5, interquartile range, 62.5–75) undergoing aortic surgery. Muscle mass was determined before and 7 days after surgery and blood samples were taken before surgery, and 1, 3, and 7 days after surgery. The circulating metabolome and proteome were determined using commercial services (Metabolon and SomaLogic).

**Results:**

Ten patients lost more than 10% of the cross‐sectional area of the rectus femoris (RF_CSA_) and were defined as wasting. Metabolomic analysis showed that 557 circulating metabolites were altered following surgery (*q* < 0.05) in the whole cohort and 104 differed between wasting and non‐wasting patients (*q* < 0.05). Weighted genome co‐expression network analysis, identified clusters of metabolites, both before and after surgery, that associated with muscle mass and function (*r* = −0.72, *p* = 6 × 10^−4^ with RF_CSA_ on Day 0, *P* = 3 × 10^−4^ with RF_CSA_ on Day 7 and *r* = −0.73, *P* = 5 × 10^−4^ with hand‐grip strength on Day 7). These clusters were mainly composed of acyl carnitines and dicarboxylates indicating that pre‐existing mitochondrial dysfunction contributes to muscle loss following surgery. Surgery elevated cortisol to the same extent in wasting and non‐wasting patients, but the cortisol:cortisone ratio was higher in the wasting patients (Day 3 *P* = 0.043 and Day 7 *P* = 0.016). Wasting patients also showed a greater increase in circulating nucleotides 3 days after surgery. Comparison of the metabolome with inflammatory markers identified by SOMAscan® showed that pre‐surgical mitochondrial dysfunction was associated with growth differentiation factor 15 (GDF‐15) (*r* = 0.79, *P* = 2 × 10^−4^) and that GDF‐15, interleukin (IL)‐8), C‐C motif chemokine 23 (CCL‐23), and IL‐15 receptor subunit alpha (IL‐15RA) contributed to metabolic changes in response to surgery.

**Conclusions:**

We show that pre‐existing mitochondrial dysfunction and reduced cortisol inactivation contribute to muscle loss following surgery. The data also implicate GDF‐15 and IL‐15RA in mitochondrial dysfunction.

## Introduction

Both chronic disease and acute injury lead to a loss of muscle mass that impacts both quantity and quality of life. It has been suggested that muscle constitutes a reservoir for amino acids, as both a source of building blocks and energy for the inflammatory response where repair is needed in non‐muscle tissue (reviewed in other studies^1^, ^2^, ^3^, ^4^). Consequently, changes in the circulating metabolome and proteome are likely to result from atrophy and inform on underlying processes.

As the loss of muscle mass is common to a wide range of acute and chronic conditions, it might be expected that the extent of muscle loss would be tightly associated with the severity of the underlying disease. However, this is not the case.^4^ For example, in chronic obstructive pulmonary disease (COPD) and chronic heart failure patients, the association of disease severity with muscle mass is found in large studies but is not necessarily observed in small studies.^5^, ^6^, ^7^ The loss of muscle mass and function also varies in acute conditions as exemplified by the response to aortic surgery, where we have found patients can lose between nothing and 40% of the cross‐sectional area of the rectus femoris in 7 days in a manner that cannot be accounted for by standard measures of disease severity or intra‐operative factors.^8^ This variability is important for two reasons: first, because reduced muscle mass and strength are associated with increased mortality, and second, in trials of anabolic agents, the variability of response increases the size of trials markedly.

One potential contributor to the variability in response is growth differentiation factor 15 (GDF‐15). In a model of muscle wasting after aortic surgery, we showed that patients who wasted had higher levels of GDF‐15 and lower levels of insulin‐like growth factor 1 (IGF‐1) in their circulation over the 7 days following surgery.^8^ Whilst GDF‐15 is predominantly thought to promote muscle loss by suppressing appetite,^9^, ^10^, ^11^ the association of GDF‐15 with muscle loss in this model (in which patients were starved prior to and in the first 18 h after surgery) together with the demonstration that over‐expression of GDF‐15 in the muscle of mice promotes local atrophy^12^ implies that GDF‐15 also has direct effects on the muscle. Such muscle loss following surgery is not limited to aortic surgery and has also been noted in patients following hip^13^ or knee replacement surgery^14^ and gastric bypass surgery^15^ as well as occurring following partial hepatectomy in live liver donors.^16^ Differences in surgical methodology can also produce different effects on muscle with laparoscopic cholecystectomy promoting a small increase in C‐reactive protein (CRP) and IL6 and no detectable loss in grip strength whereas open cholecystectomy caused a larger increase in CRP and IL6 and a demonstrable loss of grip strength.^17^

It seems likely that the circulating metabolome of patients who waste significantly will differ from those who do not. Analysis of these differences may highlight both potential biomarkers of wasting as well as offer mechanistic insight into muscle loss. Consequently, in this study, we analysed the metabolic profile of patients undergoing aortic surgery in the model we have previously published,^8^, ^18^ comparing circulating metabolites in patients who wasted significantly after surgery [loss of >10% of the cross‐sectional area of the rectus femoris, (RF_CSA_)] with those who did not waste significantly using an unbiased approach. Our data set also included significant physiological data allowing us to use the statistical methods employed in the R package weighted genome co‐expression network analysis (WGCNA) to identify clusters of metabolites and their association with physiology and with the levels of GDF‐15 measured in the circulation. Whilst this approach was developed for the unbiased identification of clusters of co‐expressed genes that form networks,^19^ it has also been used to analyse metabolomic data.^20^ Finally, we compared the metabolite profile with measurements of inflammatory mediators made using SOMAscan® that were available from a subset of the samples.

## Methods

### Patients

The patients included in this study are a subset from a previously reported cohort.^18^ The patients were undergoing elective aortic surgery at the Royal Brompton Hospital and provided written informed consent upon recruitment to the study, which had been approved by the National Research Ethics Committee (07/Q0204/68). The inclusion and exclusion criteria are given in Paul *et al*.^18^ Muscle loss was determined as reduction in RF_CSA_ between Days 0 and 7 after surgery, determined by ultrasound as previously described.^21^ Patients were divided into wasting and non‐wasting patients based on the loss of more than or less than 10% RF_CSA_, which we have previously shown is a robust detectable difference in RF_CSA_ determined by ultrasound.^8^ Physiological assessment of handgrip strength and quadriceps strength were carried out within 1 week prior to surgery as previously described.^18^ Patients were included in the study using the following selection criteria, male patients from our original study with complete muscle functional data were divided into wasting and non‐wasting patients. Those with complete plasma sample sets and transcriptomic data were identified, and an additional complete sample set with either the next greatest muscle loss or the next best muscle preservation was selected (see Supporting Information, *Figure* S1).

### Blood samples and analysis

Blood samples were taken before the induction of anaesthesia on the day of surgery (Day 0) and on Days 1, 3, and 7 after surgery into EDTA tubes, and plasma was prepared and stored at −80°C until required. The plasma samples were sent to Metabolon for metabolomic analysis and to SomaLogic for the SOMAscan® assay of 1300 proteins. All samples passed the appropriate quality control measurements.

GDF‐15 was quantified separately by enzyme linked immunosorbent assay (ELISA) according to the manufacturer's instructions (R and D Systems).

### Statistical methods

Raw counts were taken from the Metabolon output and entered into MetaboAnalyst 4.0 (https://www.metaboanalyst.ca/). Missing values were imputed by replacement with the minimum positive value for the metabolite then log transformed. Principle component analysis (PCA) was performed (Aabel™ 3.0 Gigawiz Ltd) and one sample shown to be an outlier. This sample was removed and the PCA repeated.

To allow analysis by two‐way ANOVA, individuals with missing time points were removed leaving eight non‐wasting patients and nine wasting patients.

#### Weighted genome co‐expression network analysis

To compare metabolite expression with physiological parameters (or chemokines), the data for each day from all patients except the outlier removed from the PCA were analysed using the R package WGCNA separately. Correlation coefficients between both modules and individual metabolites with physiological parameters were determined using robust biweight‐midcorrelation.

## Results

### Patient demographics

We have previously shown that we can reproducibly detect a loss of 10% RF_CSA_ by ultrasound allowing us to divide patients into wasting and non‐wasting groups.^21^ To determine differences in metabolism between patients who lost muscle following surgery and those who did not, from our initial cohort of 40 patients,^18^ we selected samples from 10 patients who lost more than 10% RF_CSA_ and 10 patients who did not lose a significant amount of muscle. The demographics and surgical parameters of this set of patients are shown in Table 1. The samples included were selected based on being male (to reduce variability because of sex), on the availability of sufficient sample from all 4 days, on the relative amount of muscle lost (to increase between group variability), and on the ability to pair samples with a previously analysed transcriptomic data set. Patients who lost muscle mass spent longer in the intensive care unit (ICU) and in the hospital and tended to spend more time on mechanical ventilation although this did not reach statistical significance. However, there were no pre‐surgical differences in age, body mass index (BMI), cardiac function (left ventricular ejection fraction, %), renal function (estimated glomerular filtration rate), and no difference in bypass time or cross‐clamp time. EuroSCORE did not differ between the two groups in the entire cohort, but in this selected cohort, EuroSCORE was higher in wasting than non‐wasting patients. In addition to the differences in quadriceps muscle size and loss following surgery, patients who lost muscle also showed greater loss of strength measured as hand‐grip strength (*Table* 1). Consistent with our previous study,^8^ we also observed a marked increase in circulating GDF‐15 following surgery and this increase was larger in patients who wasted than in those who did not waste (*Figure* S2).

**Table 1:** Physiological characteristics of aortic surgery patients

	Non‐wasting patients (*n* = 10)	Wasting patients (*n* = 10)	*P* value
**Demographic data**			
Age (year)	69(49–75)	71.5 (67–75)	ns
BMI (kg/m^2^)	27.5 ± 2.4	28.5 ± 4.9	ns
EuroSCORE 2	1.35 (1–2.7)	3.7(1.7–5.8)	0.034
Pre‐operative LVEF (%)	60.3 ± 8.9	54.2 ± 11.4	ns
Pre‐operative creatinine clearance (eGFR) (μmol/L)	73.8 ± 12.1	65.7 ± 18.5	ns
**Muscle data**			
RF_CSA_ Day 0	7.3 ± 2.1	6.4 ± 0.8	ns
RF_CSA_ Day 7	7.2 ± 2.1	5.4 ± 0.8	0.025
Change in RF_CSA_ (%)	0.8(−0.83–2.95)	14.5 (11.87–17.66)	<0.001
Hand grip d0	30.8 ± 8.2	26.1 ± 5.7	ns
Hand grip d7	30.17 ± 7.9	19.6 ± 8.1	0.011
**WHO performance status**			
Pre‐operative	0 (0–1)	1( 0–1)	ns
Post‐operative	1 (1–2)	2 (1–3)	ns
**Operative data**			
Total bypass time (min)	125.8 ± 36.5	131.2 ± 30.7	ns
Total cross‐clamp time (min)	93 ± 30	94 ± 26	ns
**Critical care data**			
ICU length of stay (days)	1 (1–1)	3 (1–7)	0.038
Hospital length of stay (days)	7 (6–8)	13.5 (9–24)	0.014
Mechanical ventilation (h)	16 (10–21)	25 (22–99.5)	0.05
Vasopressor duration (h)	36 (19.5–48)	46 (22–114)	ns

Data are presented as mean ±SD for normally distributed data or as median (interquartile range) for data that were not normally distributed. BMI, body mass index; ICU, intensive care unit; LVEF, left ventricular ejection fraction; ns, not significant; RF_CSA_, rectus femoris cross‐sectional area; WHO, World Health Organization.

## Metabolomic effects of surgery

**Figure 1:**
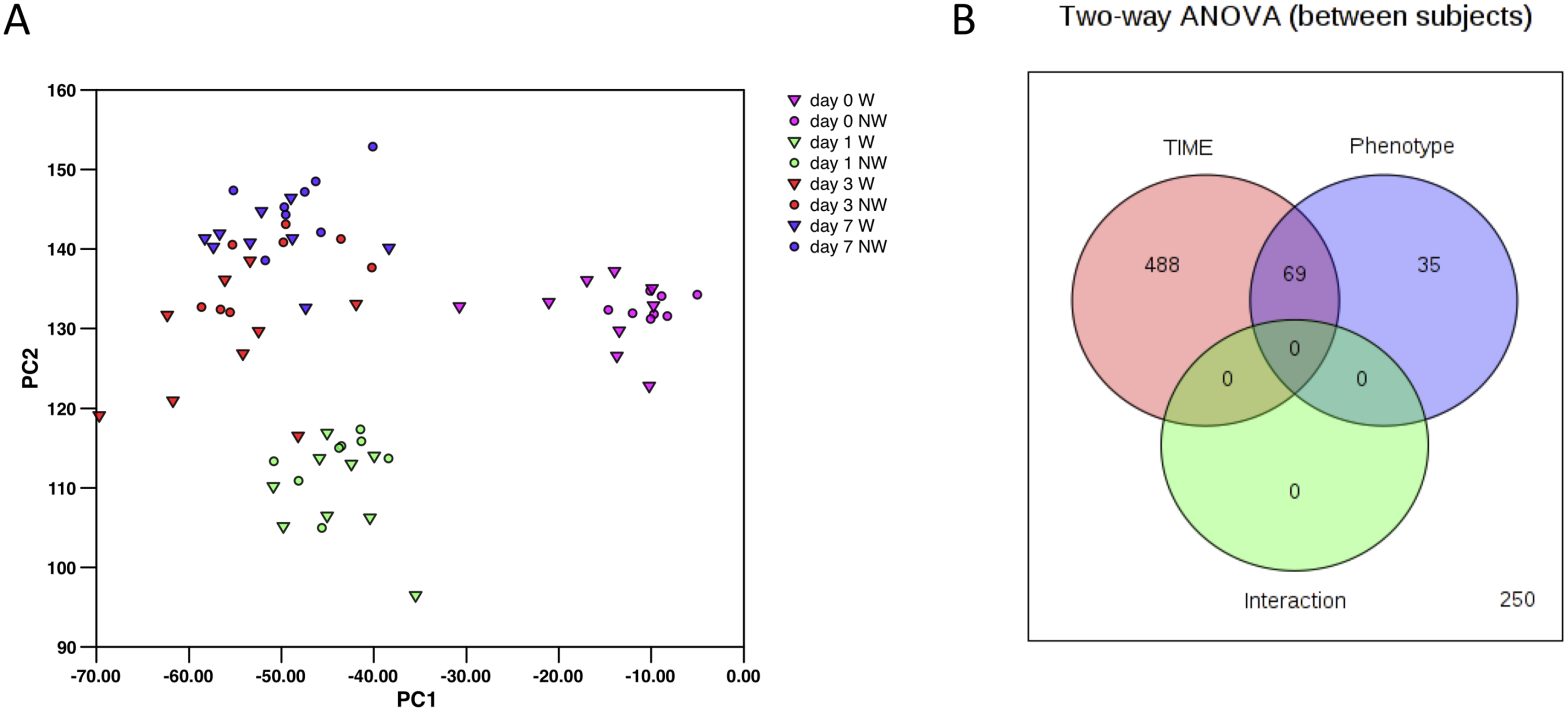
The effect of surgery on plasma metabolites in wasting and non‐wasting patients. (A) Principal component analysis of all metabolites shows clear separation of the metabolic profiles into three groups: before surgery (Day 0) 24 h post‐surgery (Day 1) and a cluster containing both Days 3 and 7 post‐surgery. (B) Two‐way ANOVA comparing the effect of time and phenotype (wasting vs. non‐wasting patients) on plasma metabolites; 488 metabolites were different as a function of time alone, 36 differed as a function of phenotype with 69 metabolites altered as a function of both time and phenotype.

Principal component analysis readily separated samples from the Days 0 and 1 with samples from Days 3 and 7 forming a large third group for the bulk of the data. However, one patient was identified as a significant outlier in the data set with their Days 0 and 1 samples associating more closely with the Day 3/7 clusters than the Days 0 and 1 cluster. Subsequently, it was confirmed that this patient required renal replacement therapy and so their samples were removed from the data set. PCA of the remaining samples showed a similar pattern with three major groups (Day 0, Day 1, and Days 3/7, *Figure* 1A).

**Figure 2:**
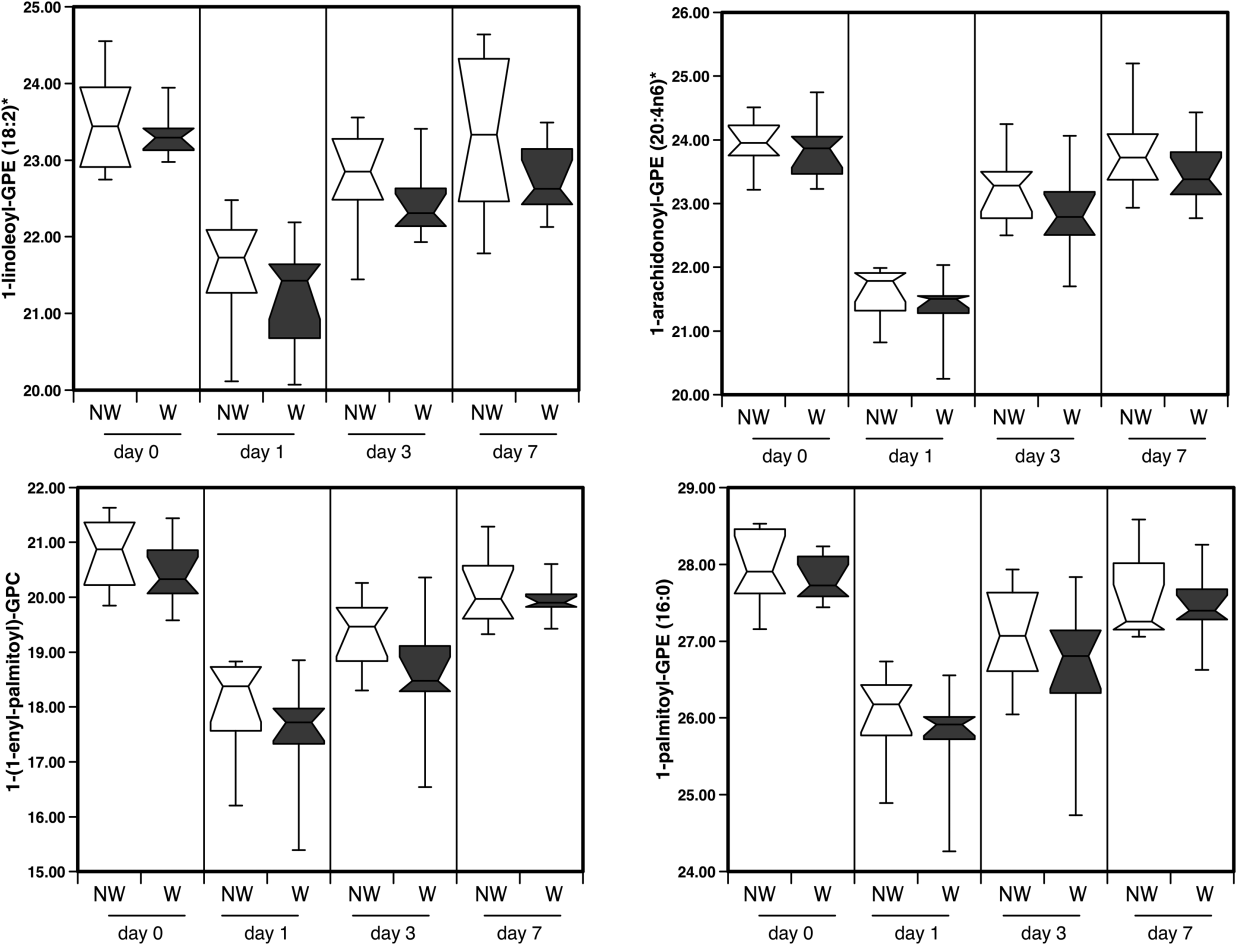
The effect of surgery on lysophospholipids and lysoplasmalogens in the circulation of wasting and non‐wasting patients. The typical profile of lysophospholipids and lysoplasmalogens is shown. These metabolites were suppressed following surgery before returning to their pre‐surgery level over the subsequent 7 days.

Analysis by two‐way ANOVA showed that 557 compounds changed significantly (*q* < 0.05) with time and 104 were different between the wasting and non‐wasting patients with 69 of these metabolites common to both data sets (*Figure* 1B). Unsurprisingly, the compounds showing the greatest change with time were drugs, with lidocaine (used at the time of taking the Day 1 biopsy) the most significantly different by time. The 557 compounds identified as showing a significant time‐dependent change included 293 lipids with 120 amino acids or their metabolites and 26 nucleotides or their metabolites (*Tables* 2 and S1 with all metabolite data given in Data S2). The predominant effect of surgery on lipid levels (216 lipids) was a reduction followed by a recovery towards or above pre‐surgery levels (*Figure* 2). Of the lipids showing this pattern, the class of lipids most altered were lysophospholipids and lysoplasmalogens, which made up over 50% of the 30 most altered lipids (arranged either by significance or by fold reduction, *Table* S1). Fifty‐six lipids showed a greater than two‐fold reduction after surgery. The effect of surgery on non‐esterified fatty acids (NEFA) was to cause a reduction on Day 1 with these lipids remaining low throughout the experiment (*Table* S1).

**Table 2:** Most significant changes in lipid, amino acid/peptide, and nucleotide metabolites with respect to time

Metabolite	Pathway/type	*P* (adjusted)	Fold change Day 1	Fold change Day 3	Fold change Day 7
Lipids					
1‐Linoleoyl‐GPE (18:2)*	Lysophospholipid	6.0 E‐19	0.196	0.657	1.030
1‐Arachidonoyl‐GPE (20:4n6)*	Lysophospholipid	5.7 E‐17	0.203	0.628	0.938
1‐Oleoyl‐GPE (18:1)	Lysophospholipid	3.5 E‐16	0.230	0.651	1.117
Nervonoylcarnitine (C24:1)*	Acyl carnitine	4.1 E‐15	0.533	1.159	1.895
1‐Palmitoyl‐2‐arachidonoyl‐GPI (16:0/20:4)*	Phosphatidylinositol	9.9 E‐15	0.422	1.050	1.615
1‐Palmitoyl‐GPE (16:0)	Lysophospholipid	1.8 E‐13	0.306	0.675	0.943
1‐(1‐Enyl‐palmitoyl)‐GPC (P‐16:0)*	Lysoplasmalogen	2.5 E‐13	0.168	0.372	0.744
Stearoylcarnitine (C18)	Acyl Carnitine	9.4 E‐13	0.439	0.837	1.132
Ximenoylcarnitine (C26:1)*	Acyl Carnitine	2.2 E‐12	0.618	1.177	2.100
2‐Palmitoyl‐GPC (16:0)*	Lysophospholipid	2.3 E‐12	0.250	0.525	0.807
Amino acids					
N‐Acetylputrescine	Polyamine	1.5 E‐16	4.53	1.87	1.35
Pro‐hydroxy‐pro	Proline	2.4 E‐13	16.03	5.59	1.91
Gamma‐glutamylisoleucine*	Gamma‐glutamyl amino acid	2.3 E‐12	0.59	1.57	1.61
5‐Hydroxyindole sulfate	Tryptophan	3.4 E‐12	0.14	0.32	0.08
Glutamine	Glutamate	4.1 E‐11	0.71	0.68	0.70
Phenylacetylglycine	Acetylated peptides	1.0 E‐10	4.40	16.70	14.56
Methionine sulfoxide	Methionine	1.2 E‐10	2.11	2.52	1.64
Isoleucylglycine	Dipeptide	2.1 E‐10	17.49	7.17	2.69
6‐Oxopiperidine‐2‐carboxylate	Lysine	2.6 E‐10	0.96	3.17	3.65
Trans‐4‐hydroxyproline	Proline	2.6 E‐10	2.96	2.34	1.30
Nucleotides					
Uridine	Pyrimidine	5.42E‐17	0.27	0.42	0.89
Xanthine	Purine	1.06E‐08	0.36	0.34	0.56
Allantoin	Purine	3.29E‐08	0.70	0.70	0.80
5,6‐Dihydrouracil	Pyrimidine	1.23E‐05	0.50	0.80	1.03
Urate	Purine	2.01E‐05	0.73	0.73	0.82
5‐Methyluridine (ribothymidine)	Pyrimidine	2.92E‐05	0.65	0.62	0.74
Beta‐alanine	Pyrimidine	1.18E‐04	1.07	1.21	1.78
2′‐O‐methyluridine	Pyrimidine	1.51E‐04	1.24	1.64	2.07
N‐Acetyl‐beta‐alanine	Pyrimidine	2.40E‐04	1.45	1.11	1.60
2′‐O‐Methylcytidine	Pyrimidine	3.42E‐04	2.46	3.11	4.24

A two‐way ANOVA was performed on log‐transformed data in Metaboanalyst as described in Methods. Fold change was calculated on unlogged data. C(x) refers to carbon chain length; GPC, glycerol phosphsphorylcholine; GPE, glycerol phosphsphorylethanolamine;

* metabolite identification by mass spectrum only.

**Figure 3:**
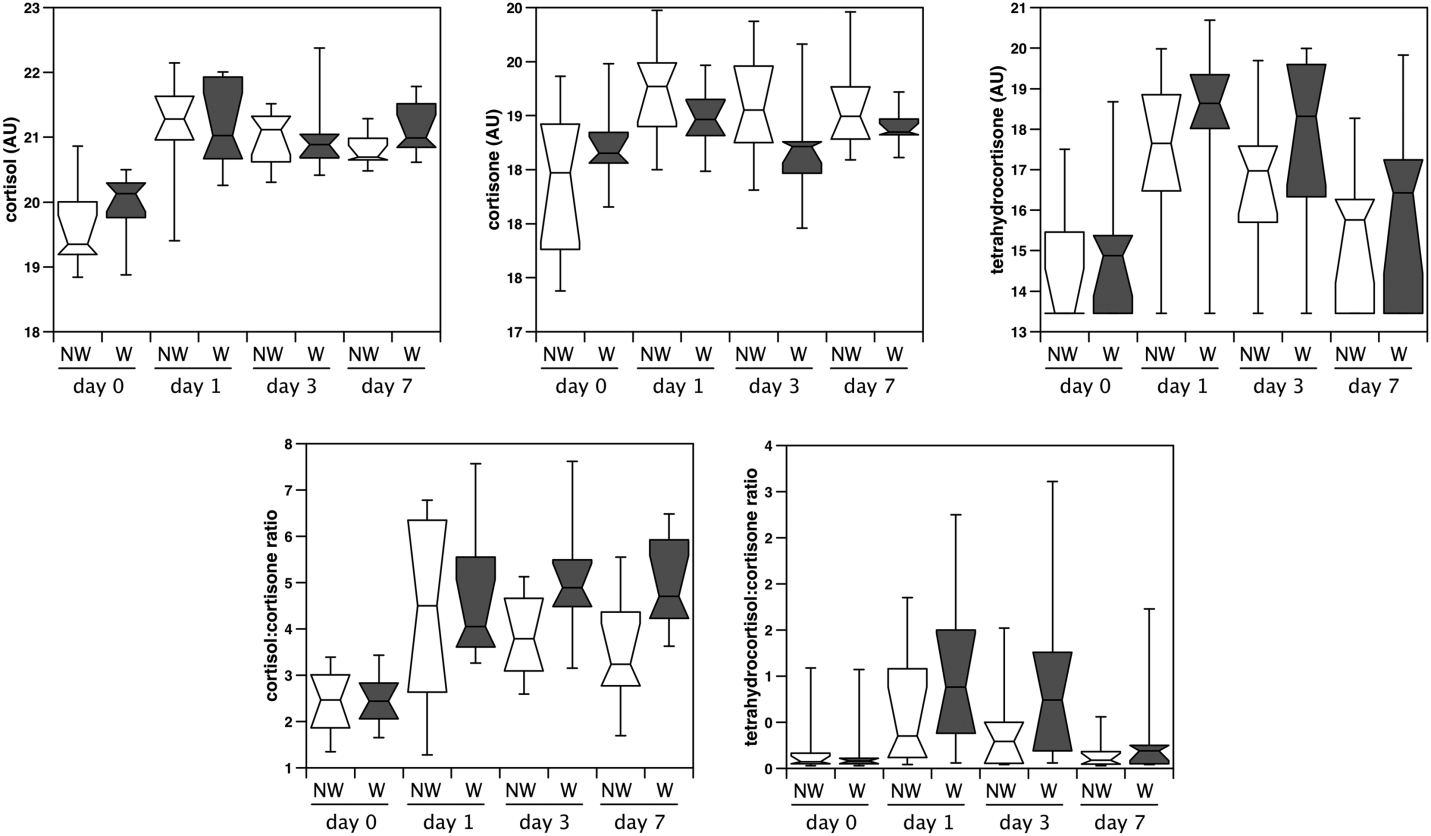
The effect of surgery on cortisol metabolites in wasting and non‐wasting patients. Cortisol, cortisone, and tetrahydrocortisol were quantified in the plasma of patients undergoing aortic surgery. Data were normalized as described in Methods. To calculate the ratios, the raw data were used. Cortisol increased in both wasting patients and non‐wasting patients to a similar extent. Cortisone increased significantly in the non‐wasting patients but not in the wasting patients. Conversely, median tetrahydrocortisol was higher in wasting than in non‐wasting patients. Consequently, the cortisol to cortisone ratio was higher in wasting patients at the end of the period. The tetrahydrocortisol to cortisone ratio also tended to be higher in wasting than non‐wasting patients although this difference did not reach significance.

Of the lipids that increased on the first day after surgery, the lipid showing the greatest increase was tetrahydrocortisol sulfate, which increased 17‐fold (*Table* 3 and *Figure* 3). Consistent with this observation, both cortisol and cortisone were increased following surgery (*Figure* 3). Interestingly, cortisol levels were similar between the two groups and showed the same increase and trend. In contrast, cortisone levels increased in the non‐wasting patients between Days 0 and 1 remaining high throughout the experiment, but in the wasting patients, cortisone levels did not increase suggesting a potential difference in cortisol metabolism between the two groups. The cortisol:cortisone ratio was lower in the wasting patients than the non‐wasting patients on Days 3 and 7 (*P* = 0.043 and *P* = 0.016, respectively) whereas although the tetrahydrocortisone:cortisone ratio was higher in wasting patients than non‐wasting patients on Days 1 and 3, these differences did not reach statistical significance (*Figure* 3).

**Table 3:** Lipids elevated more than three‐fold on Day 1 after surgery compared with Day 0 arranged by fold increase

Metabolite	Pathway/type	*P* (adjusted)	Fold change Day 1	Fold change Day 3	Fold change Day 7
Tetrahydrocortisol sulfate	Corticosteroids	7.3E‐05	17.69	14.16	3.00
17alpha‐Hydroxypregnanolone glucuronide	Pregnenolone steroids	7.0E‐03	15.16	7.25	4.40
Beta‐sitosterol	Sterol	2.2E‐03	11.21	8.25	3.28
Estrone 3‐sulfate	Estrogenic steroids	8.9 E‐07	10.99	12.10	1.50
Palmitoyl‐arachidonoyl‐glycerol (16:0/20:4)^1^ *	Diacylglycerol	2.9 E‐02	7.90	2.54	24.28
5alpha‐Pregnan‐diol disulfate	Progestin steroids	1.0 E‐03	7.01	2.41	1.22
2‐hydroxyadipate	Dicarboxylate	4.1 E‐02	5.70	4.33	6.54
Adipoylcarnitine (C6‐DC)	Acyl carnitine	1.4 E‐06	5.04	4.52	3.25
Taurocholate	Primary Bile Acid	7.2 E‐07	4.68	345.54	194.49
Suberoylcarnitine (C8‐DC)	Acyl carnitine	2.7 E‐04	3.99	4.40	3.33
Pregnenolone sulfate	Pregnenolone steroids	1.8 E‐04	3.57	1.08	1.01
Arachidonoylcholine	Acyl choline	2.3 E‐03	3.54	2.69	11.21
3,4‐Methyleneheptanoylcarnitine	Acyl carnitine	4.2 E‐02	3.49	1.72	1.38
Myo‐inositol	Inositol	3.6 E‐09	3.23	1.94	1.07
Pregnenediol sulfate (C21H34O5S)*	Pregnenolone steroids	8.6 E‐05	3.08	1.44	0.79
Taurodeoxycholate	Bile acid	6.8 E‐06	3.05	111.00	126.14
Cortisol	Corticosteroids	1.5 E‐08	3.00	2.50	2.49

A two‐way ANOVA was performed on log‐transformed data in Metaboanalyst as described in Methods. Fold change was calculated on unlogged data. C(x) refers to carbon chain length; DC, dicarboxylate;

* metabolite identification by mass spectrum only.

**Figure 4:**
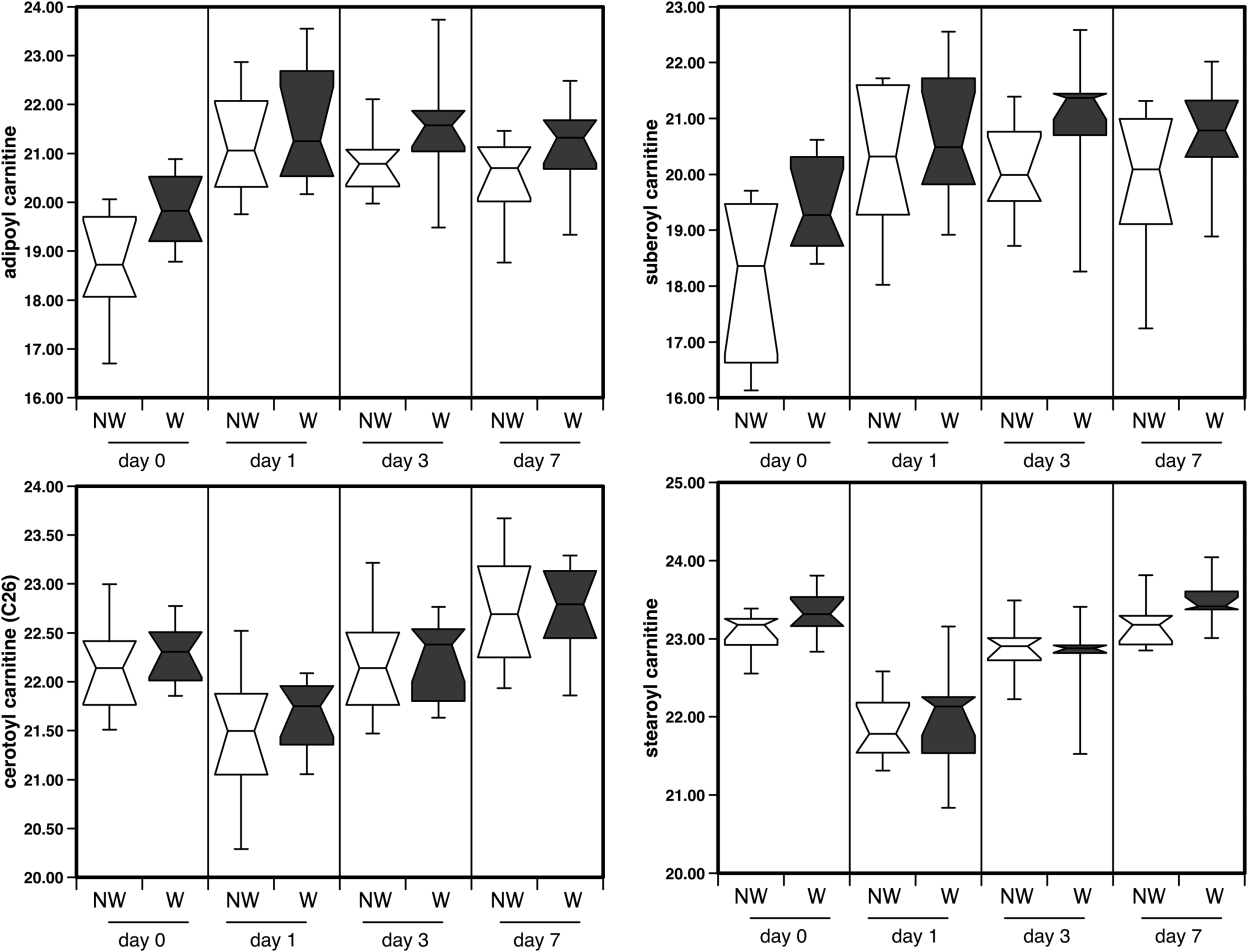
The effect of surgery on short‐chain and long‐chain acyl carnitines in the circulation of wasting and non‐wasting patients. Short chain and dicarboxylate acyl carnitines were increased following surgery and remain elevated especially in wasting patients whereas long‐chain acyl carnitines were suppressed following surgery then returned to baseline values within 7 days.

A number of acyl carnitines were elevated by surgery; these included the short‐chain fatty acyl carnitines (C < 14) and the acyl carnitine derivatives of fatty acid dicarboxylates of all chain lengths. However, with the exception of octadecanedioyl carnitine all the long‐chain acyl carnitines (C ≥ 14) were reduced on the first day after surgery (*Table* 4). Neither the short‐chain acyl carnitines nor the acyl carnitine derivatives of fatty acid dicarboxylates returned to baseline by Day 7, remaining elevated. Conversely, the long‐chain acyl carnitines did not remain depressed and returned towards their baseline level or exceeded it by 7 days after surgery (*Figure* 4, *Table* 4).

**Table 4:** Changes in plasma acyl carnitine derivatives with respect to time following surgery

Acyl carnitine	*P* (adjusted)	Fold change Day 1	Fold change Day 3	Fold change Day 7
Adipoylcarnitine (C6‐DC)	1.4E‐06	5.04	4.52	3.25
Suberoylcarnitine (C8‐DC)	2.7E‐04	3.99	4.40	3.33
3,4‐Methyleneheptanoylcarnitine	4.2E‐02	3.49	1.72	1.38
Decanoylcarnitine (C10)	2.3E‐03	1.95	1.84	2.49
Octanoylcarnitine (C8)	9.0E‐03	1.84	1.48	1.85
Octadecenedioylcarnitine (C18:1‐DC)*	1.7E‐02	1.76	1.96	1.70
Hexanoylcarnitine (C6)	7.0E‐03	1.75	1.55	1.52
cis‐4‐Decenoylcarnitine (C10:1)	2.0E‐02	1.65	1.48	1.83
Pimeloylcarnitine/3‐Methyladipoylcarnitine (C7‐DC)	2.0E‐02	1.57	2.65	2.92
Octadecanedioylcarnitine (C18‐DC)*	1.8E‐03	1.53	1.76	1.91
Laurylcarnitine (C12)	1.1E‐03	1.30	1.49	2.13
Myristoleoylcarnitine (C14:1)*	1.9E‐03	0.88	1.21	1.59
Behenoylcarnitine (C22)*	3.1E‐06	0.78	1.47	2.31
Myristoylcarnitine (C14)	6.1E‐07	0.67	1.06	1.33
Lignoceroylcarnitine (C24)*	2.1E‐11	0.67	0.95	1.52
Cerotoylcarnitine (C26)*	7.3E‐07	0.67	1.04	1.50
Ximenoylcarnitine (C26:1)*	2.2E‐12	0.62	1.18	2.10
Margaroylcarnitine (C17)*	9.6E‐08	0.57	0.79	1.14
Nervonoylcarnitine (C24:1)*	4.1E‐15	0.53	1.16	1.89
Palmitoleoylcarnitine (C16:1)*	3.4E‐06	0.51	0.77	1.12
Oleoylcarnitine (C18:1)	9.0E‐12	0.47	0.80	1.10
Palmitoylcarnitine (C16)	2.8E‐11	0.46	0.79	1.07
Eicosenoylcarnitine (C20:1)*	1.9E‐07	0.45	1.43	1.87
Linoleoylcarnitine (C18:2)*	1.7E‐07	0.45	0.61	0.94
Stearoylcarnitine (C18)	9.4E‐13	0.44	0.84	1.13
Dihomo‐linoleoylcarnitine (C20:2)*	1.7E‐06	0.41	0.72	1.13
Arachidoylcarnitine (C20)*	2.6E‐10	0.41	0.72	1.15
Linolenoylcarnitine (C18:3)*	4.0E‐09	0.39	0.54	0.86
Arachidonoylcarnitine (C20:4)	1.5E‐10	0.32	0.44	0.85

A two‐way ANOVA was performed on log‐transformed data in Metaboanalyst as described in Methods. Fold change was calculated on unlogged data. C(x) refers to carbon chain length; DC, dicarboxylate;

* metabolite identification by mass spectrum only.

## Wasting vs. non‐wasting

Amino acids were the class of the metabolite that showed the most significant difference in profile between wasting and non‐wasting patients (*Tables* 5 and S2). Unsurprisingly, the majority of these amino acids increased more in wasting patients than in the those that did not waste. The amino acids showing the most significant changes were N‐acetylated amino acids. As there is no known mechanism in humans for N‐acetylation of free amino acids but the majority of proteins become N‐acetylated at or shortly after synthesis,^22^, ^23^ the presence of N‐acetylated amino acids in circulation is likely to be an indicator of catabolism. Similarly, nucleotides increased in the circulation more in wasting than non‐wasting patients again consistent with catabolic processes. Both the amino acids and nucleotides tended to peak on Day 3 after surgery suggesting that maximal catabolism occurred around this time (*Tables* 5 and S2). There were few differences in xenobiotics between wasting and non‐wasting patients (*Table* S2). The xenobiotics showing the greatest differences between these groups were morphine and its glucuronide metabolite, which were both higher in wasting patients than in non‐wasting patients. However, there was no association of morphine or morphine–glucuronide with muscle loss on Day 1 when levels of these compounds were highest.

**Table 5:** Metabolites most significantly different between patients who waste and those who do not

Metabolite	Pathway/type	*P* (adjusted)	W/NW d1	W/NW d3	W/NW d7
**Lipids**						
Methylmalonate	Fatty acid	1.6 E‐04	1.44	1.32	1.36	
3‐Hydroxybutyrylcarnitine	Acyl carnitine	6.1 E‐04	1.03	0.97	1.18	
1‐(1‐Enyl‐palmitoyl)‐2‐linoleoyl‐GPE (P‐16:0/18:2)*	Plasmalogen	1.4 E‐03	0.91	0.58	0.84	
Acetylcarnitine (C2)	Acyl carnitine	2.2 E‐03	0.96	0.97	1.41	
1‐Stearoyl‐2‐linoleoyl‐GPI (18:0/18:2)	Phosphatidylinositol	6.1 E‐03	1.19	1.16	1.49	
2‐Aminooctanoate	Fatty acid, amino	7.4 E‐03	0.89	1.14	0.98	
1‐Stearoyl‐2‐linoleoyl‐GPC (18:0/18:2)*	Phosphatidylcholine	7.4 E‐03	1.03	1.08	1.15	
Malonylcarnitine	Acyl carnitine	7.9 E‐03	0.98	0.70	1.46	
1,2‐Dilinoleoyl‐GPC (18:2/18:2)	Phosphatidylcholine	7.9 E‐03	1.05	1.13	1.27	
3‐Methylglutarate/2‐methylglutarate	Fatty acid, dicarboxylate	9.6 E‐03	0.93	0.60	1.13	
**Amino acids**						
N‐Acetyl‐1‐methylhistidine*	Histidine	4.7 E‐05	2.12	3.24	4.16	
N‐Acetylcitrulline	Arginine/proline	1.6 E‐04	2.10	1.70	1.35	
N‐Acetylglutamine	Glutamate	1.9 E‐04	1.26	1.32	1.14	
N‐Acetylphenylalanine	Phenylalanine	5.1 E‐04	1.37	1.84	1.32	
3‐Methylglutarylcarnitine	branched chain amino acid	1.4 E‐03	1.59	4.67	2.26	
N‐Acetylarginine	Arginine/proline	1.6 E‐03	1.87	1.56	1.27	
Glutarylcarnitine (C5‐DC)	Lysine	1.6 E‐03	1.42	2.15	1.14	
Gamma‐glutamylmethionine	Gamma‐glutamyl amino acid	1.6 E‐03	0.89	0.91	0.75	
N‐Acetylleucine	branched chain amino acid	2.5 E‐03	1.29	1.13	1.24	
Hydroxyasparagine*	Alanine/aspartate	2.5 E‐03	1.37	1.82	1.25	
N‐Acetylserine	Glycine/serine/threonine	2.7 E‐03	1.48	1.85	1.17	
**Nucleotides**						
5,6‐Dihydrouridine	Pyrimidine	7.4 E‐03	1.27	2.09	1.35	
Orotidine	Pyrimidine	7.4 E‐03	1.50	2.46	1.23	
Cytidine	Pyrimidine	7.4 E‐03	0.80	1.20	0.86	
Dihydroorotate	Pyrimidine	9.6 E‐03	1.72	1.21	1.25	

A two‐way ANOVA was performed on log‐transformed data in Metaboanalyst as described in Methods. Fold change at each time point for each individual was calculated on unlogged data. The average fold change for each metabolite in wasting patients was then divided by the average fold change in non‐wasting patients at the same time point to give W/NW. C(x) refers to carbon chain length; DC dicarboxylate; GPC, glycerol phosphsphorylcholine; GPE, glycerol phosphsphorylethanolamine; GPI, glycerol phosphsphorylinositol;

* metabolite identification by mass spectrum only.

### Associations of metabolite levels with clinical phenotype

To explore the data further, we used the WGCNA package to identify clusters of metabolites present on each day. Robust biweight‐midcorrelation analysis was then used to identify associations between these clusters or the metabolites they contained and clinical phenotype [muscle size (RF_CSA_), RF_CSA_ loss, strength (hand‐grip), strength loss, and GDF‐15 level]. WGCNA grouped the metabolites into 22 clusters on Day 0, 19 clusters on Day 1, 12 clusters on Day 3, and 12 clusters on Day 7 (*Figures* S3–S6). As the content of each cluster differs on each day but they are automatically labelled, in the following description, the clusters are distinguished with the superscript representing the day.

#### Pre‐surgical metabolites, phenotype, and growth differentiation factor 15

Comparison of the clusters present on Day 0 with physiology and pre‐surgical GDF‐15 levels indicated that two clusters (light‐green^0^ and cyan^0^) had the strongest associations with muscle mass and function and with circulating GDF‐15 (*Figure* S3). Of these, light‐green^0^ was most tightly associated with muscle parameters (*r* = −0.72, *P* = 6 × 10^−4^ with RF_CSA_ on Day 0, *r* = −0.74, *P* = 3 × 10^−4^ with RF_CSA_ on Day 7 and *r* = −0.73, *P* = 5 × 10^−4^ with hand‐grip strength on Day 7), and cyan^0^ most tightly associated with circulating GDF‐15 (*r* = 0.79, *P* = 2 × 10^−4^).

The light‐green^0^ cluster contains predominantly the acyl carnitines whilst the cyan^0^ cluster contains predominantly fatty acid dicarboxylates (*Table* S3). Indeed, comparing the metabolites with muscle mass and to a lesser extent strength, prior to surgery, showed that fatty acid dicarboxylates and their acyl‐carnitine derivatives were more strongly negatively associated with muscle cross‐sectional area than their monocarboxylate derivatives (*Table* S4). Fatty acid dicarboxylates and their carnitine metabolites were also markedly enriched in the metabolites associated with pre‐surgical levels of GDF‐15 as well as with hand‐grip strength 7 days after surgery (*Table* S5). These dicarboxylates are produced by omega oxidation of fatty acids and can be increased as a response to starvation, an excess hepatic fat or as a consequence of diabetes.^24^

#### Days 1 and 3 metabolites with phenotype and growth differentiation factor 15

Comparison of the Days 1 and 3 clusters with physiology again demonstrated strong associations between individual modules [on Day 1 with red^1^ and turquoise^1^ (*Table* S6), on Day 3 with turquoise^3^ (*Table* S7)] with muscle size and strength both before surgery and 7 days after surgery (*Figures* S4 and S5). Both turquoise modules were also strongly associated with circulating GDF‐15 levels (*Figures* S4 and S5).

Red^1^ is predominantly lysophospholipids, whereas both turquoise^1^ and turquoise^3^ are enriched for amino acids and nucleotides and contain the acyl carnitines and the fatty acid dicarboxylates. On both days, the associations of the modules were stronger with the post‐surgical than pre‐surgical physiological measurements. Further, analysis of the associations with GDF‐15 showed that it was the amino acid and nucleotide components of the module that had the strongest association with the growth factor (*Table* S8).

#### Day 7 metabolites with phenotype and growth differentiation factor 15

On Day 7 the circulating metabolome did not associate with muscle size or strength either at the beginning or at the end of the study. However, one module (black^7^) was strongly associated with the loss of hand‐grip strength (*r* = 0.79, *P* = 3 × 10^−4^_,_ *Figure* S6). This module contained many xanthine metabolites, consistent with increased purine metabolism and a loss of adenosine triphosphat (ATP) (*Table* 6). Consistent with all other days, circulating GDF‐15 on Day 7 was strongly correlated with the turquoise^7^ module (*r* = 0.85, *P* = 7 × 10^−5^); again, this module was enriched for amino acids, nucleotides, and their metabolites, and GDF‐15 was associated with circulating levels of these types of metabolites (Table S9).

**Table 6:** Metabolites in the plasma on Day 7 correlated with loss of grip strength over the 7 days

Metabolite	Pathway/type	Module	*r*	*P*
Paraxanthine	Xanthine metabolism	black	0.83	3.0E‐05
1,7‐Dimethylurate	Xanthine metabolism	black	0.82	6.2E‐05
1,3‐Dimethylurate	Xanthine metabolism	black	0.81	7.1E‐05
5.Acetylamino‐6‐amino‐3‐methyluracil	Xanthine metabolism	black	0.80	1.1E‐04
3‐Methyl‐catechol‐sulfate	Benzoate metabolism	black	0.76	4.0E‐04
5‐Acetylamino‐6‐formylamino‐3‐methyluracil	Xanthine metabolism	black	0.76	4.5E‐04
1,3,7‐Trimethylurate	Xanthine metabolism	black	0.75	4.9E‐04
Theophylline	Xanthine metabolism	black	0.75	6.0E‐04
Sucrose	Disaccharides and oligosaccharides	black	0.74	6.3E‐04
Caffeic acid sulfate	Xanthine metabolism	black	0.74	7.0E‐04
3‐Hydroxypyridine sulfate	Chemical	black	0.72	1.1E‐03
Theobromine	Xanthine metabolism	black	0.71	1.3E‐03
Catechol sulfate	Benzoate metabolism	black	0.71	1.3E‐03
1‐(1‐Enyl‐stearoyl)‐2‐arachidonoyl‐GPE (P‐18:0/20:4)	Plasmalogen	blue	0.71	1.5E‐03
Quinate	Food component/plant	black	0.69	2.1E‐03
Cytidine	Pyrimidine metabolism, Cytidine containing	blue	−0.68	2.6E‐03
1‐Methylxanthine	Xanthine metabolism	black	0.68	2.6E‐03
N‐Palmitoylserine	Endocannabinoid	red	0.68	2.7E‐03
Caffeine	Xanthine metabolism	black	0.68	2.7E‐03
Guaiacol sulfate	Benzoate metabolism	black	0.68	2.9E‐03
1‐Methylurate	Xanthine metabolism	black	0.66	3.7E‐03
Trigonelline (N′‐methylnicotinate)	Nicotinate and nicotinamide metabolism	black	0.66	4.0E‐03
Mannose	Fructose, mannose, and galactose metabolism	black	−0.64	5.2E‐03
Perfluorooctanesulfonate	Chemical	green	−0.63	6.2E‐03
3‐Methylxanthine	Xanthine metabolism	black	0.63	6.4E‐03
Atorvastatin lipitor	Xenobiotics	black	−0.63	6.6E‐03
1‐(1‐Enyl‐palmitoyl)‐2‐Arachidonoyl‐GPE (P‐16:0/20:4)	Plasmalogen	blue	0.63	6.8E‐03
Atorvastatin lactone	Xenobiotics	black	−0.62	7.7E‐03
3‐Hydroxybutyrylcarnitine	Fatty acid metabolism(acyl carnitine)	blue	−0.61	8.7E‐03
2,3‐Dihydroxyisovalerate	Food component/plant	black	0.61	8.8E‐03
7‐Methylurate	Xanthine metabolism	black	0.61	9.6E‐03

GPE, glycerol phosphorylethanolamine.

#### Metabolic phenotype and inflammatory proteins

The associations of GDF‐15 may be direct or be a consequence of the induction of GDF‐15 by a factor that also drove the changes in metabolite concentration. The majority of the cohort (17 of 19) from which we obtained the metabolic data was part of a larger study investigating the circulating proteome response to surgery using SOMAscan® that we will report elsewhere. However, as inflammation forms a significant part of the response to surgery, we investigated whether any inflammatory markers from the CC or CXC chemokine families identified in the analysis were stronger associates with the circulating metabolome on Day 1 after surgery. Consistent with our analysis of GDF‐15 by ELISA, GDF‐15 was strongly associated with the turquoise^s1^ module (*r* = 0.75, *P* = 5 × 10^−4^), which was enriched for amino acids and nucleotides (*Table* S10). Several chemokines or receptors showed similar positive associations with the turquoise^s1^ module (*Figure* S7); these included interleukin‐8 (IL‐8) (CXCL8, *r* = 0.76, *P* = 4 × 10^−4^), C‐C motif chemokine 23 (CCL‐23) (*r* = 0.71, *P* = 0.001), and IL‐15 receptor subunit alpha (IL‐15RA) (*r* = 0.74, *P* = 8 × 10^−4^), whereas interleukin‐5 (IL‐5) had a negative association with the same module (*r* = −0.8, *P* = 1 × 10^−4^). Together, these data suggest that a range of inflammatory‐signalling factors may contribute to increased muscle loss in this condition either directly stimulating muscle loss, promoting the release of factors that promote muscle wasting, or promoting the recruitment of inflammatory cells to promote muscle catabolism in response to surgery. However, in the Day 0 samples, GDF‐15 showed the strongest association with the turquoise^s0^ (*Figure* S8) module, which was enriched for acetylated fatty acids and fatty acid dicarboxylates (*Table* S11).

## Discussion

Our analysis identifies both general trends in the metabolic response of individuals to surgery (defined as all procedures in preparation for surgery and in the immediate post‐surgical maintenance of the patient, in addition to the surgical procedure itself) as well as particular differences associated with either the loss of muscle mass or function. Together, they provide a picture where there is a significant loss of lipids from the circulation, and that differences in cortisol metabolism and mitochondrial function prior to surgery contribute to the differences in the muscle response of individuals to this insult. We will deal briefly with the general consequences and then focus on changes associated with changes in muscle mass and function as these were the focus of this study.

### General changes

There are several likely drivers of changes in metabolites that will occur in all patients. The first is the potential for an early starvation response as the patients starved prior to surgery (mostly overnight) and remain starved for a significant period after the operation. The second is a direct response in the level of metabolites released from damaged tissue. Third, there is the potential for the effects of the range of pharmacological agents used to anaesthetise and stabilize the physiology of the patients during surgery as well as for post‐surgical analgesia. Finally, there are potential effects of the cardiopulmonary bypass process itself, including inflammation, the ischemia‐reperfusion response that will arise from the application and subsequent release of the aortic cross clamp and any interactions of the blood with oxygenator membranes and other exogenous surfaces. These factors will drive the early changes in metabolome and will contribute to the later metabolic profile as will the inactivity and other pharmacological interventions required by the period of time spent in the ICU and high‐dependency unit. Other than the presence of drugs in the circulation, the most striking general trend is a reduction in the levels of many circulating lipids including lysophospholipids and NEFA. The early response of mammals to starvation is characterized by glycogenolysis and gluconeogenesis, with lipids released from stores increasing plasma NEFA. This response was demonstrated in a recent metabolomic study of starvation in healthy human subjects that showed a marked increase in circulating NEFA in the first 2 days of starvation.^25^ This response is opposite to the reduction in these metabolites that we see here, suggesting that any starvation response is masked by other factors. Other studies of patients undergoing bypass surgery have also shown reduced circulating lipids.^26^, ^27^ One possible cause for this loss is the loss of the lipids in the oxygenator membrane, a suggestion supported by the observation that Day 1 is the lowest measured value for the majority of these lipids.

### Differences between wasting and non‐wasting patients

The metabolic changes that differentiate wasting and non‐wasting patients can be classified into those that identify potential biomarkers of wasting and those that provide mechanistic information.

#### Potential biomarkers of wasting

The metabolites that are potential circulating biomarkers of a wasting process include amino acids and nucleotides that most likely represent the breakdown of muscle tissue and loss of ATP. Consistent with these molecules representing increased muscle breakdown, rather than reflecting tissue damage directly caused by the surgery, they were increased following surgery to a greater extent in the patients who wasted than in those who did not. Prominent in the amino acids were the methyl derivatives of histidine, consistent with the use of 3‐methyl‐histidine as a marker of elevated muscle breakdown. Furthermore, circulating creatinine was higher in wasting patients than in those who did not waste. Similarly, there is evidence of increased plasma purines and other nucleotide derivatives. The increased purines (uric acid, xanthine, and hypoxanthine) are often used as markers of oxidative stress.^28^, ^29^ However, in this case, it is also possible that they represent loss of ATP (and other nucleotides) as part of tissue loss and turnover. The amounts of these metabolites in circulation tend to peak at or around Day 3 suggesting that this is the period of maximal protein degradation.

### Potential mechanistic markers

Two sets of metabolites that differed between wasting and non‐wasting patients perhaps provide some mechanistic information with one set suggesting differences in cortisol metabolism and the other indicating a role for mitochondrial dysfunction in muscle wasting.

#### Cortisol metabolism

Unsurprisingly, in all patients, there was a marked increase in circulating concentrations of cortisol following surgery. Circulating cortisol did not differ between wasting and non‐wasting patients, but there was no significant increase in cortisone in the plasma of patients who wasted whereas cortisone increased markedly in non‐wasting patients. Cortisol is a potent signal for muscle wasting,^30^ raising the possibility that differences in cortisol metabolism could account for part of the difference in muscle wasting. Cortisol conversion to cortisone is catalyzed by 11‐β‐hydroxysteroid dehydrogenase 2 (11‐βHSD2) and to tetrahydrocortisone by the 5α and 5β‐cortisol reductases.^31^, ^32^ The higher cortisol:cortisone ratio in the wasting patients than in the non‐wasting patients in the presence of a similar increase in cortisol suggests that overall 11‐βHSD2 levels are lower in the wasting patients. Previous studies have shown that the increase in cortisol in response to critical illness is only partly due to increased cortisol production and that reduced cortisol metabolism and clearance contributes to the residual increase.^33^ The proposed mechanism reducing 11‐βHSD2 and cortisol reductase activity was increased circulating bile acids and their metabolites acting as inhibitors. However, whilst bile acids increased following surgery, the majority did not show a significant difference between wasting and non‐wasting patients, and those that did were higher in the non‐wasting patients indicating that they were not responsible for the difference in cortisol metabolism. It is possible therefore that intrinsic differences in the relative activity of 11‐βHSD2, 11‐βHSD1, and the cortisol reductases contribute to the variation in metabolism seen in these patients. Such differences may be caused by genetic differences affecting expression or activity, such as the polymorphisms in 11‐βHSD2 that associate with hypertension,^34^ or epigenetic variation, as studies in rats and in humans have shown differential methylation of the 11‐βHSD2 locus is associated with hypertension.^35^, ^36^, ^37^ Whilst the examples given affect 11‐βHSD2 activity, genetic and epigenetic variation at other loci may also contribute to differences in cortisol metabolism.

#### Mitochondrial dysfunction

Two of the major sets of metabolites (fatty acid dicarboxylates and the acyl carnitine derivatives) were (i) elevated by surgery and (ii) associated with reduced muscle mass and loss of strength. Conversion of fatty acids into their acetyl carnitine derivatives allow them to be transported into mitochondria for β‐oxidation. As the generation of the acetyl carnitine derivatives is not rate limiting in the oxidation of fatty acids and occurs outside mitochondria, acyl carnitines can accumulate in cells with reduced mitochondrial activity and from there enter the circulation. Consequently, increased circulating acyl‐carnitine levels have been used as a marker of mitochondrial dysfunction.^38^ For example, increased acyl carnitines have been shown in response to liver damage caused by paracetamol overdose but not to liver damage caused by furosemide.^38^ Increased acyl carnitines have been reported in patients with heart failure^39^ in which their levels are associated with a number of disease parameters including N‐terminal pro‐brain natriuretic peptide and urea^40^ as well as predicting cardiovascular mortality.^41^ Increased acyl carnitines are also present in the circulation of patients with critical illness and again associated with mitochondrial dysfunction.^42^ In this case, levels correlated positively with mitochondrial DNA in the circulation. Dicarboxylate fatty acids are also markers of oxidative stress and damage that occurs in response to mitochondrial dysfunction. Like the acyl carnitines, increased circulating fatty acid dicarboxylates are present in heart failure^39^, ^43^ as well as in the metabolic syndrome.^44^ The carnitine derivatives of these dicarboxylates have also been shown to be predictive of increased mortality in patients following coronary artery bypass grafting.^45^ Our data therefore indicate that mitochondrial dysfunction is an important component of the maintenance of muscle function in disease and with the loss of muscle mass and function following surgery.

The observation that mitochondrial dysfunction is associated with an increased potential for muscle loss in patients with critical illness is consistent with a number of other studies both in animals and in man. In mouse models of sepsis, there is a marked reduction in mitochondrial mass and function as demonstrated by a reduction in metabolic rate.^46^ Similarly, patients with multi‐organ failure who survive show higher levels of PGC1α and better maintenance of complex I activity, indicative of maintained mitochondrial function.^47^ In a long‐term rat model of sepsis, reduced complex I activity and ATP depletion were also associated with mortality.^48^ Two studies have indicated that mitochondrial dysfunction as a consequence of the co‐morbidities of the patient prior to critical illness is associated with muscle loss and/or weakness. First, in the early phase of critical illness Puthucheary *et al*. showed a greater reduction in muscle ATP levels and loss of mitochondrial function in patients with significant co‐morbidities prior to the period of critical illness and these patients lost more muscle than those with better preserved mitochondrial function.^49^ Second, our study of miRNAs in this cohort of patients showed that pre‐surgical miR‐542‐3p was proportional to the amount of muscle lost in patients and that elevated expression of this miRNA caused mitochondrial dysfunction by disrupting mitochondrial ribosome formation.^50^ That expression of this miRNA is inversely proportional to left ventricular ejection fraction in our aortic surgery patients and to transfer capacity for carbon monoxide (TL_CO_) in COPD patients provides a potential mechanism by which the primary disease may promote muscle mitochondrial dysfunction to reduce energy usage in the muscle.^50^, ^51^

Comparing the circulating proteome with the metabolome both before and after surgery suggests potential regulators of the changes in metabolism. This analysis suggests a role for both GDF‐15, IL‐15, and IL‐8 in the metabolic response to surgery. GDF‐15 is an anorexogenic hormone that inhibits appetite by activating the GFRAL receptor in the hippocampus.^10^ At face value, therefore the associations of GDF‐15 with metabolome may be through reduced food intake. However, this may not be the case for the association of the metabolome on Day 1 after surgery, as both the metabolome levels and GDF‐15 levels have changed, but all the patients remain unfed. Furthermore, the association with amino acids in circulation on Day 3 is consistent with a direct role for circulating GDF‐15 on muscle protein turnover. We have previously shown that over‐expression of GDF‐15 in the tibialis anterior of mice leads to atrophy of the GDF‐15 expressing muscle (10% loss in 14 days) but no loss in the contra‐lateral control.^12^ Furthermore, we have shown GDF‐15 can increase transforming growth factor‐β (TGF‐β) activated kinase 1 (TAK1) activity, in muscle cells in culture^52^ in the absence of any SMAD response suggesting that these effects are independent of contaminating TGF‐ β1, which has previously been shown in commercial preparations of GDF‐15. These data together with the association of GDF‐15 with muscle size and function but not BMI in COPD and pulmonary arterial hypertension patients imply that GDF‐15 can signal directly in the muscle. However, the signalling mechanism involved remains to be fully established. We also cannot rule out the possibility that the associations occur in our patients because of direct or indirect associations of GDF‐15 with other signalling factors. To investigate this problem in our patients, we also compared the circulating metabolome with levels of all chemokines identified by the Somalogic assay as well as with GDF‐15 in the same assay. This analysis confirmed the ELISA analysis for GDF‐15 but also identified IL‐8, CCL‐23, and IL‐15RA as strongly associated with plasma metabolome. These cytokines and receptor are all elevated in response to the surgical insult. Of these cytokines/cytokine receptors, IL‐15RA has previously been shown to directly affect oxidative metabolism. In humans, polymorphisms in IL‐15 and IL‐15RA are associated with elite athletic performance,^53^ and in mice, deletion of IL‐15RA leads to increased fatigue resistance and an increase in muscle mitochondrial density,^54^ a phenotype that is similar to that of mice that over‐express IL‐15,^55^ raising the possibility that IL‐15RA inhibits IL‐15 signalling in muscle. IL‐15RA is a high‐affinity IL‐15 binding protein that lacks intrinsic kinase activity but has been suggested to be either a component of a tripartite receptor complex or to present IL‐15 to other cells for signalling. The tissue source of the circulating IL‐15RA is not known, but the receptor can be synthesized both in a membrane bound and soluble form. This soluble form of IL‐15RA acts as an inhibitor of circulating IL‐15 suggesting that reduced IL‐15 signalling may account for some of the loss of mitochondrial function following surgery.

### Limitations of the study

Our study identifies significant changes in the metabolism of patients following aortic surgery some of which appear to distinguish patients who lose muscle from those who do not. Furthermore, it suggests that mitochondrial dysfunction and differential cortisol metabolism are important components of this process. However, the study is correlative in nature and although it is supported by other studies, this study does not establish causation. Our model of muscle loss associated with critical illness is specific to the patient group we have investigated who spend a relatively short time in intensive care. Extrapolation of our findings to a wider critical illness population therefore requires significant care.

The are many factors that lead to muscle loss on the ICU, added to which patients are in a critical condition and have probably started to lose muscle mass prior to their entry to the unit, making quantifying absolute muscle loss difficult. The extent of muscle loss is likely to be dependent on the cause of critical illness (e.g. sepsis, trauma, or surgery), the severity of the illness, duration of inactivity, and associated pharmacotherapy. For example, differences in the size or type of the event can be seen in the greater loss of strength in patients that have an open rather than laparoscopic cholecystectomy.^17^ In this study, patients undergoing open surgery had a larger acute phase response than those undergoing the laparoscopic intervention. In our study, the similarities in surgery facilitate a focus on contributions of the pre‐surgical phenotype and/or the length of critical care stay to muscle loss. Consistent with this suggestion, both pre‐surgical levels of GDF‐15 and length of stay were greater in the patients who wasted than those who did not, suggesting that both the pre‐morbid condition and time on the ICU promote muscle loss. Consistent with a role for the pre‐morbid condition, in the full cohort of patients studied here, we have shown that expression of miR‐424^56^ and miR‐542^50^ in the quadriceps, prior to surgery, are directly proportional to muscle loss following surgery. These miRNAs, which suppress protein synthesis and mitochondrial function, are associated with muscle mass and function in COPD patients and in older individuals.^50^, ^51^, ^56^ Furthermore, both miR‐542 and 424 are markedly elevated in patients with established ICUAW. Similarly, Puthucheary *et al*. showed that loss of ATP was a critical component of muscle loss and that this preferentially occurred in patients with pre‐existing chronic disease.^49^ The data in this paper showing pre‐existing mitochondrial dysfunction is associated with the loss of muscle mass and function add another consistent layer of information to these observations.

In conclusion, our data indicate that in response to surgery, there is a complex metabolic response with marked proteolysis leading to an increase in free amino acids and nucleotides that are detectable in the circulation. The extent of loss of muscle is associated with this increase in free amino acids. More importantly, our data suggest that differences in cortisol metabolism and in pre‐surgical mitochondrial function are important factors in determining the extent of muscle loss in response to this inflammatory insult.

## Funding

This work was supported by GSK and the National Institute for Health Research (NIHR) Respiratory Disease Biomedical Research Unit at the Royal Brompton and Harefield NHS Foundation Trust and Imperial College London who wholly funded RP.

## Conflicts of Interest

P.K. reports personal fees from GSK, outside the submitted work. M.G. reports grants, personal fees, and non‐financial support from GSK, personal fees from BI, personal fees from Silence therapeutics, personal fees from Cell catapult, outside the submitted work; all other authors have no conflicts of interest.

## Ethical approvals

Each clinical study was approved by the appropriate ethics committee. The appropriate study number is 07/Q0204/68. Written informed consent was obtained from all participants in the studies.

## Author contributions

The overall study was designed by P.K., A.R., and M.G. Patients were recruited and samples were collected by R.P. The laboratory studies were performed by R.P. P.K. wrote the first draft of the paper, and all authors provided critical appraisal and input into the manuscript.

## Supporting information


**Figure S1**. Flow diagram showing the selection of samples from the entire cohort
**Figure S2**. The effect of surgery on GDF‐15 in the circulation of wasting and non‐wasting patients.
**Figure S3**. Whole genome co‐expression network analysis (WGCNA) of plasma metabolites before surgery.
**Figure S4**. Whole genome co‐expression network analysis (WGCNA) of plasma metabolites 1 day after surgery.
**Figure S5**. Whole genome co‐expression network analysis (WGCNA) of plasma metabolites 3 days after surgery.
**Figure S6**. Whole genome co‐expression network analysis (WGCNA) of plasma metabolites 7 days after surgery.
**Figure S7**. Whole genome co‐expression network analysis (WGCNA) of plasma metabolites 1 day after surgery compared to plasma cytokines on the same day and their receptors.
**Figure S8**. Whole genome co‐expression network analysis (WGCNA) of plasma metabolites before surgery compared to plasma cytokines on the same day and their receptors.

**Data S1**. Excel file containing the following tables:
**Table S1**. Metabolites that differ (p<0.05) with time identified by 2‐way ANOVA
**Table S2**. Metabolites that differ (p<0.05) with phenotype identified by 2‐way ANOVA
**Table S3**. Metabolites in light green and cyan clusters day 0
**Table S4**. Metabolites in plasma before surgery that associate with RF_CSA_ before surgery
**Table S5**. Metabolites in plasma before surgery that associate with GDF‐15 before surgery
**Table S6**. Metabolites in the red and turquoise modules on day 1
**Table S7**. Metabolites in the turquoise modules on day 3
**Table S8**. Metabolites correlated with GDF‐15 on day 3
**Table S9**. Metabolites correlated with GDF‐15 on day 7
**Table S10**. Metabolites in the turquoise module on day 1 in the subset of samples analysed by SOMAscan® assay
**Table S11**. Metabolites in the turquoise module on day 0 in the subset of samples analysed by SOMAscan® assay

**Data S2**. Normalised metabolite levels for all samples
