# Supplementary material for: Metabolic profiling shows pre‐existing mitochondrial dysfunction contributes to muscle loss in a model of ICU‐acquired weakness
Source: J Cachexia Sarcopenia Muscle. 2020 Jul 16;11(5):1321–35. doi: 10.1002/jcsm.12597 (PMC7567140; doi:10.1002/jcsm.12597)
Supplement: Supplementary file 1 — Figure S1. Flow diagram showing the selection of samples from the entire cohort Figure S2. The effect of surgery on GDF‐15 in the circulation of wasting and non‐wasting patients. Figure S3. Whole genome co‐expression network analysis (WGCNA) of plasma metabolites before surgery. Figure S4. Whole genome co‐expression network analysis (WGCNA) of plasma metabolites 1 day after surgery. Figure S5. Whole genome co‐expression network analysis (WGCNA) of plasma metabolites 3 days after surgery. Figure S6. Whole genome co‐expression network analysis (WGCNA) of plasma metabolites 7 days after surgery. Figure S7. Whole genome co‐expression network analysis (WGCNA) of plasma metabolites 1 day after surgery compared to plasma cytokines on the same day and their receptors. Figure S8. Whole genome co‐expression network analysis (WGCNA) of plasma metabolites before surgery compared to plasma cytokines on the same day and their receptors. [file JCSM-11-1321-s001.pdf]

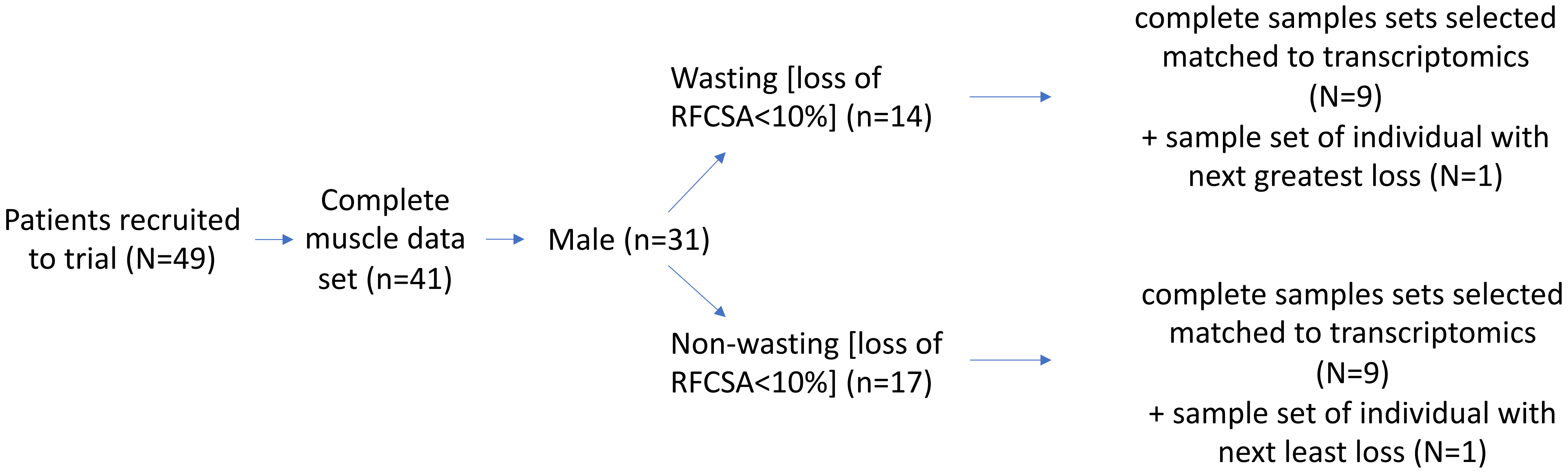

Kemp et al Fig S1

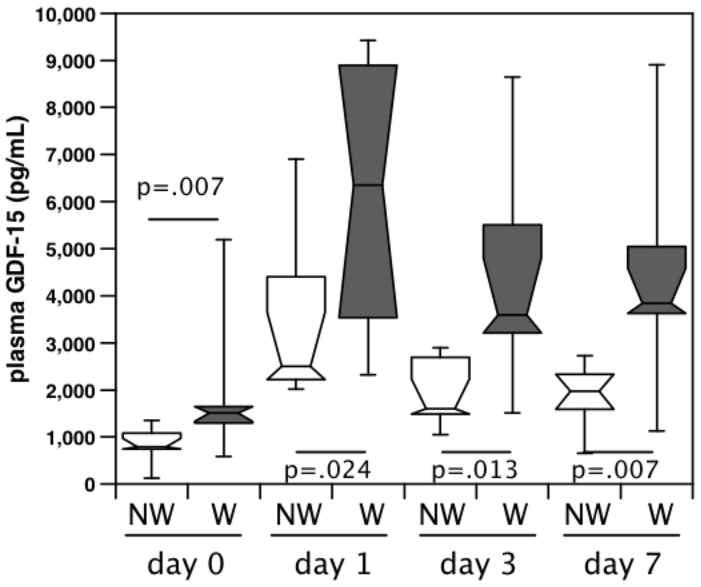

Figure S2. The effect of surgery on GDF-15 in the circulation of wasting and non-wasting patients.
Surgery increased GDF-15 in both wasting and non-wasting patients with maximal GDF-15 found on day 1.
Circulating GDF-15 was higher at all time points in wasting patients compared to non-wasting patients .

Figure S2

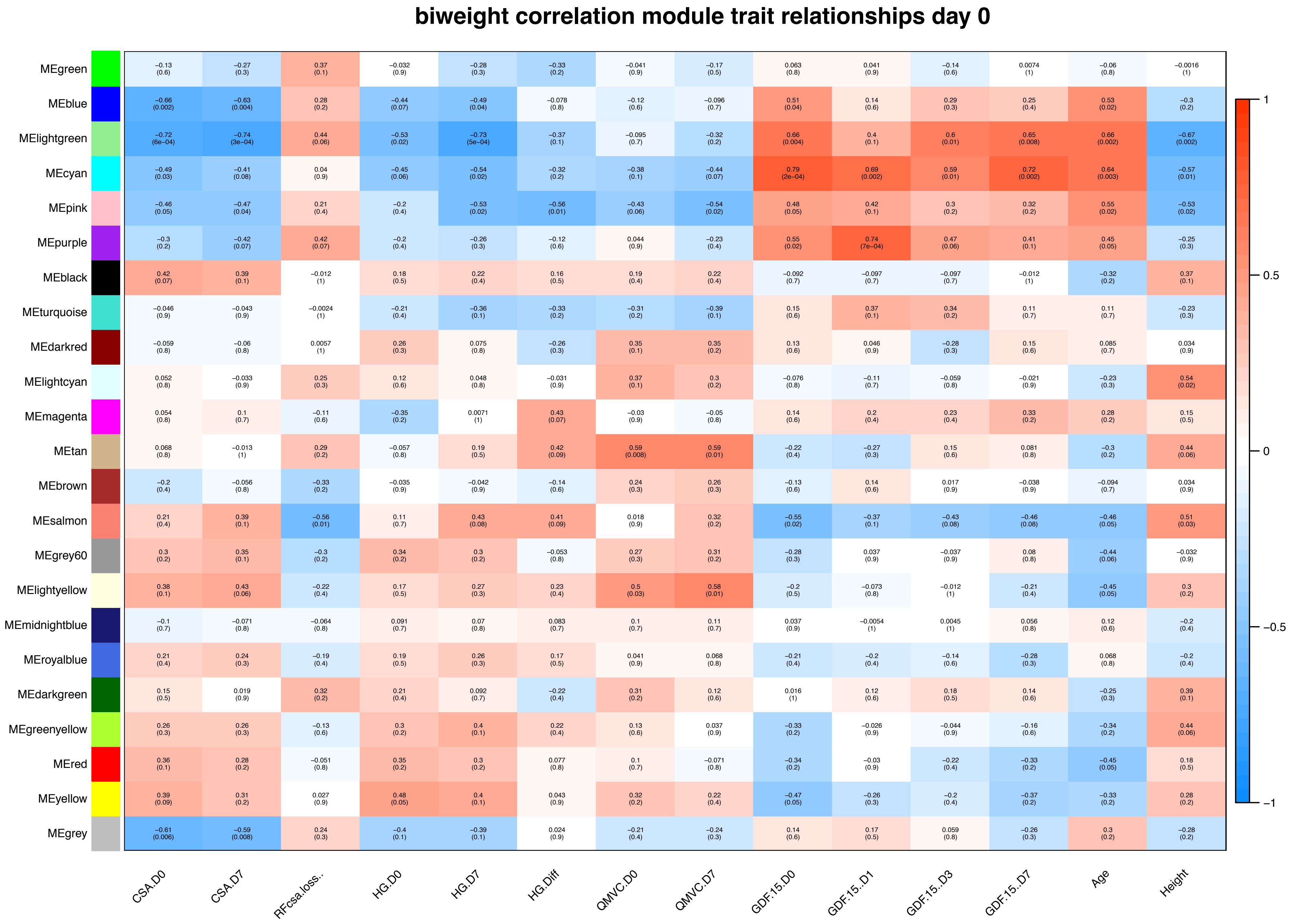

Figure S3. Whole genome co-expression network analysis (WGCNA) of plasma metabolites before surgery. Day 0 plasma metabolite levels were grouped using WGCNA then compared with physiological parameters and circulating GDF-15 levels (quantified by ELISA. Red demonstrates positive correlation and blue shows negative correlation. R and p Values calculated using robust biweight mid-correlation are shown in the appropriate box.

Figure S3

biweight correlation module trait relationships day 1

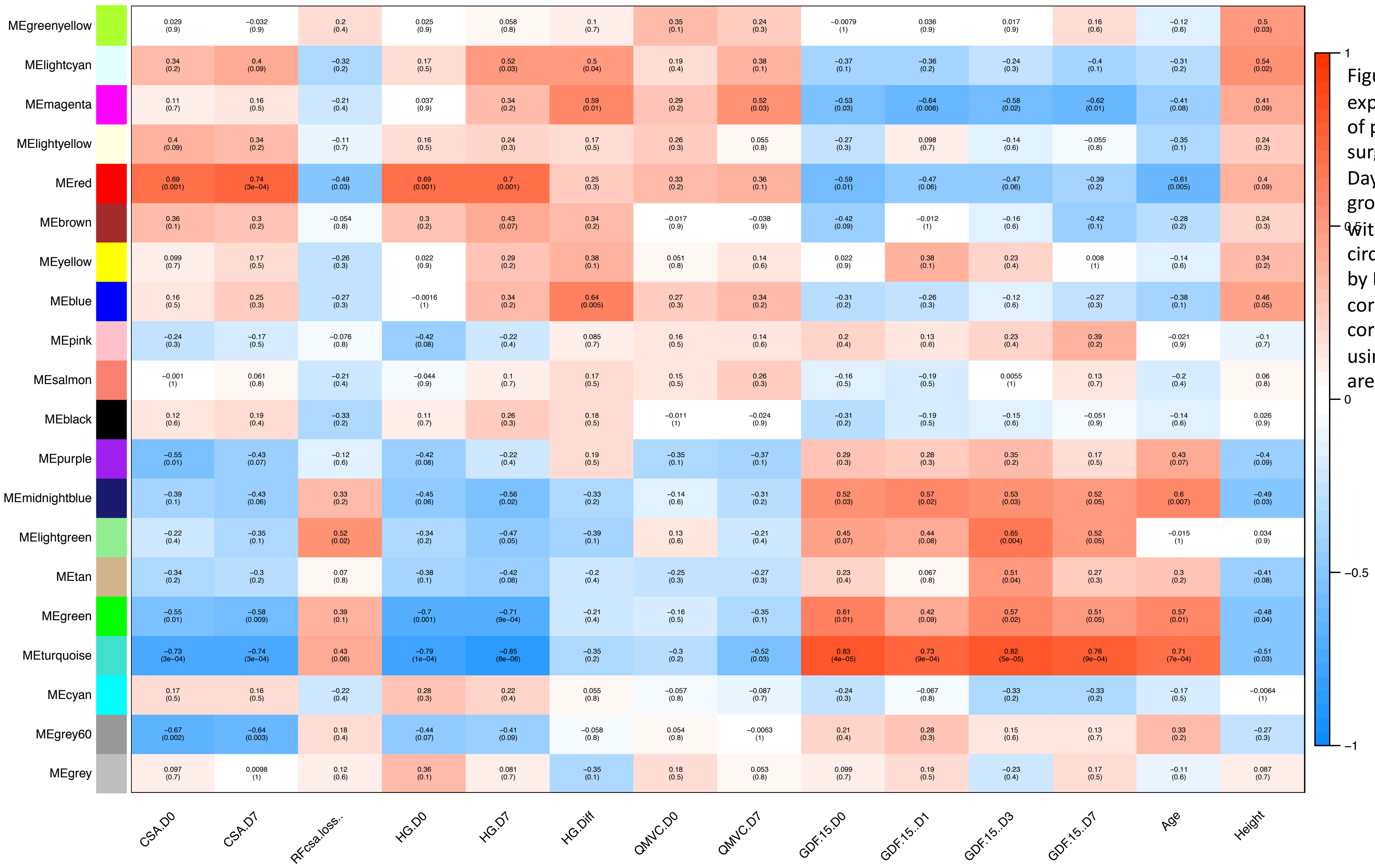

Figure S4. Whole genome co-expression network analysis (WGCNA) of plasma metabolites 1 day after surgery.
Day 1 plasma metabolite levels were grouped using WGCNA then compared with physiological parameters and circulating GDF-15 levels (quantified by ELISA. Red demonstrates positive correlation and blue shows negative correlation. R and p Values calculated using robust biweight mid-correlation are shown in the appropriate box.

Figure S4

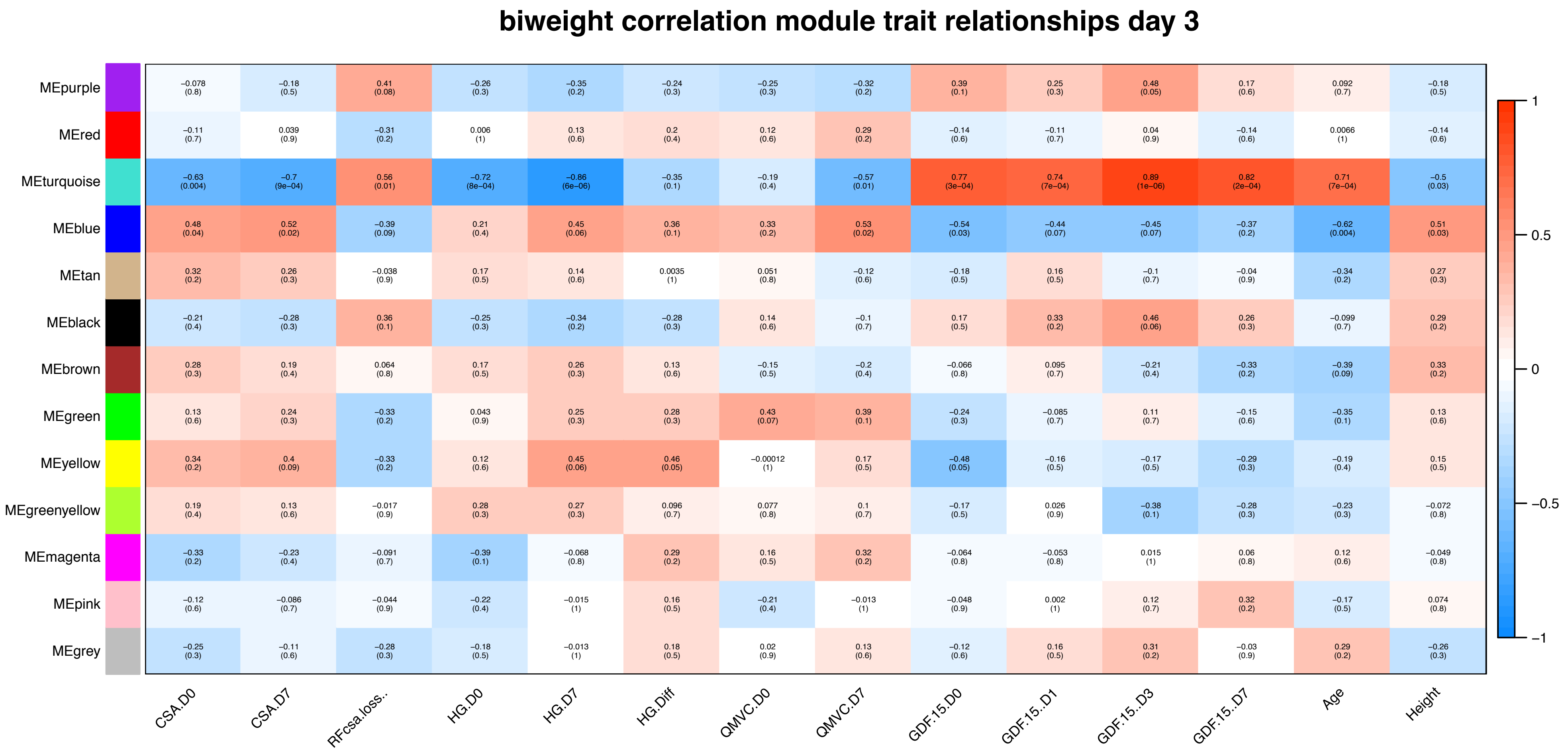

Figure S5. Whole genome co-expression network analysis (WGCNA) of plasma metabolites 3 days after surgery.
Day 3 plasma metabolite levels were grouped using WGCNA then compared with physiological parameters and circulating GDF-15 levels (quantified by ELISA. Red demonstrates positive correlation and blue shows negative correlation. R and p Values calculated using robust biweight mid-correlation are shown in the appropriate box.

Figure S5

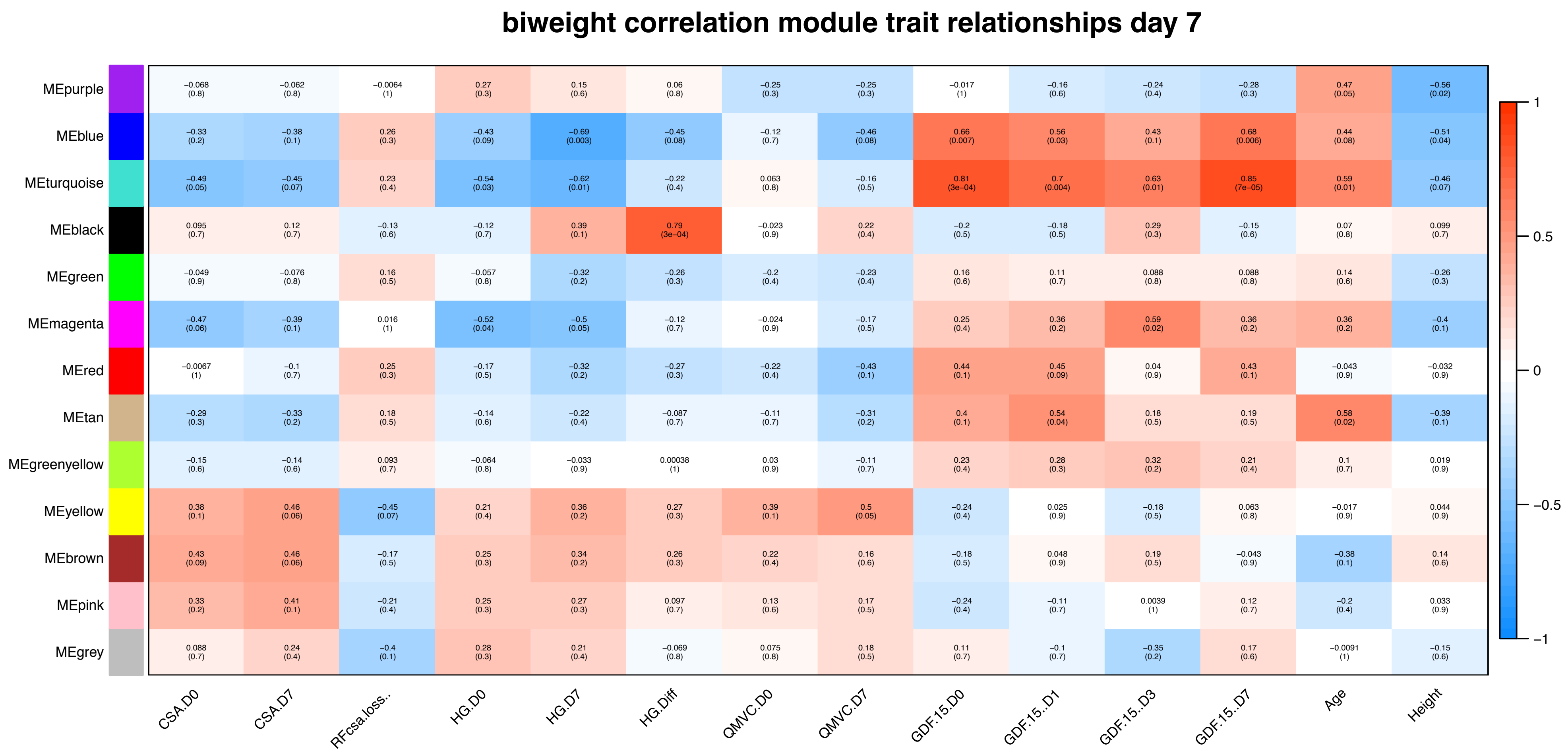

Figure S6. Whole genome co-expression network analysis (WGCNA) of plasma metabolites 7 days after surgery.
Day 7 plasma metabolite levels were grouped using WGCNA then compared with physiological parameters and circulating GDF-15 levels (quantified by ELISA. Red demonstrates positive correlation and blue shows negative correlation. R and p Values calculated using robust biweight mid-correlation are shown in the appropriate box.

Figure S6

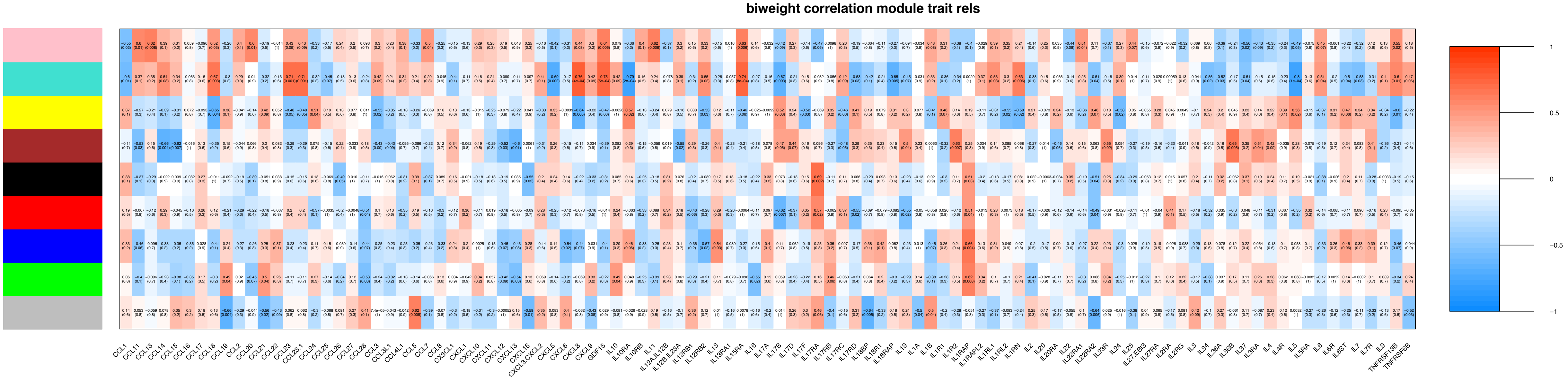

Figure S7. Whole genome co-expression network analysis (WGCNA) of plasma metabolites 1 day after surgery compared to plasma cytokines on the same day and their receptors.

Day 1 plasma metabolite levels were grouped using WGCNA then compared with circulating cytokines quantified by Somascan assay.. Red demonstrates positive correlation and blue shows negative correlation. R and p Values calculated using robust biweight mid-correlation are shown in the appropriate box.

Figure S7

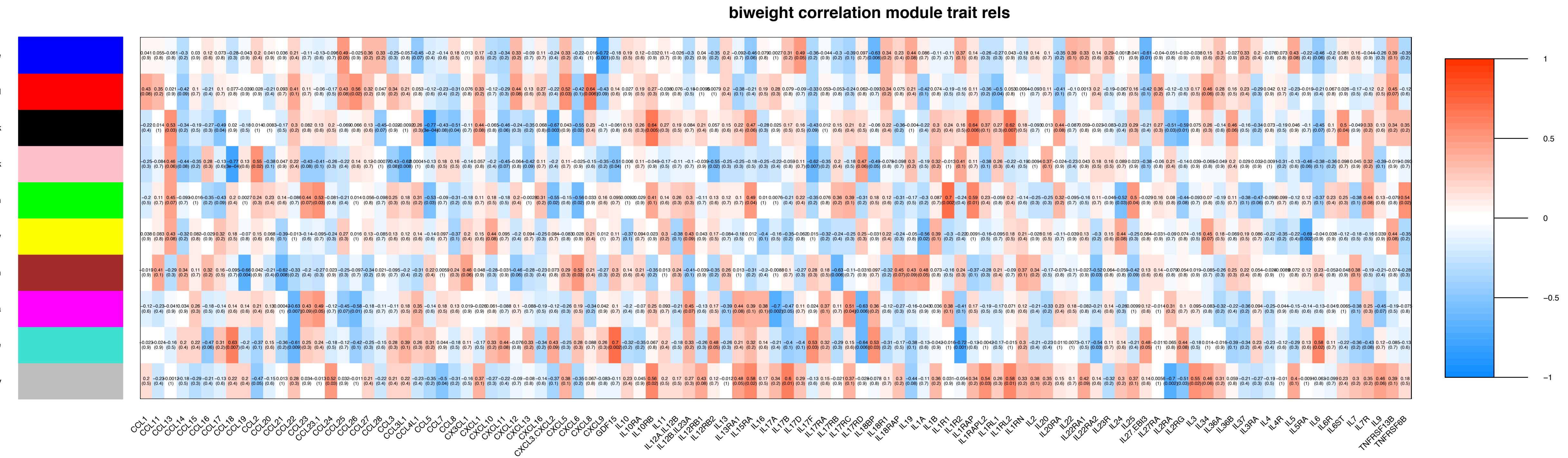

Figure S8. Whole genome co-expression network analysis (WGCNA) of plasma metabolites before surgery compared to plasma cytokines on the same day and their receptors.

Day 0 plasma metabolite levels were grouped using WGCNA then compared with circulating cytokines quantified by Somascan assay.. Red demonstrates positive correlation and blue shows negative correlation. R and p Values calculated using robust biweight mid-correlation are shown in the appropriate box.

Figure S8
